# Human milk metagenome: a functional capacity analysis

**DOI:** 10.1186/1471-2180-13-116

**Published:** 2013-05-25

**Authors:** Tonya L Ward, Sergey Hosid, Ilya Ioshikhes, Illimar Altosaar

**Affiliations:** 1 Department of Biochemistry, Microbiology and Immunology; and Ottawa Institute of Computational Biology and Bioinformatics, University of Ottawa, Ottawa, ON, K1H 8M5, Canada; 2 Ottawa Institute of Systems Biology, University of Ottawa, Ottawa, ON, K1H 8M5, Canada

**Keywords:** Human milk, Microbiome, Metagenome, Bacteria, Illumina, DNA, Open reading frames, Immune-modulatory motifs, Infant feces

## Abstract

**Background:**

Human milk contains a diverse population of bacteria that likely influences colonization of the infant gastrointestinal tract. Recent studies, however, have been limited to characterization of this microbial community by 16S rRNA analysis. In the present study, a metagenomic approach using Illumina sequencing of a pooled milk sample (ten donors) was employed to determine the genera of bacteria and the types of bacterial open reading frames in human milk that may influence bacterial establishment and stability in this primal food matrix. The human milk metagenome was also compared to that of breast-fed and formula-fed infants’ feces (n = 5, each) and mothers’ feces (n = 3) at the phylum level and at a functional level using open reading frame abundance. Additionally, immune-modulatory bacterial-DNA motifs were also searched for within human milk.

**Results:**

The bacterial community in human milk contained over 360 prokaryotic genera, with sequences aligning predominantly to the phyla of Proteobacteria (65%) and Firmicutes (34%), and the genera of *Pseudomonas* (61.1%), *Staphylococcus* (33.4%) and *Streptococcus* (0.5%). From assembled human milk-derived contigs, 30,128 open reading frames were annotated and assigned to functional categories. When compared to the metagenome of infants’ and mothers’ feces, the human milk metagenome was less diverse at the phylum level, and contained more open reading frames associated with nitrogen metabolism, membrane transport and stress response (*P* < 0.05). The human milk metagenome also contained a similar occurrence of immune-modulatory DNA motifs to that of infants’ and mothers’ fecal metagenomes.

**Conclusions:**

Our results further expand the complexity of the human milk metagenome and enforce the benefits of human milk ingestion on the microbial colonization of the infant gut and immunity. Discovery of immune-modulatory motifs in the metagenome of human milk indicates more exhaustive analyses of the functionality of the human milk metagenome are warranted.

## Background

The benefits of human milk compared to the use of commercial infant formulas are largely realized because of its bioactive components, including prebiotics, immune proteins and the microbiome of human milk itself. Breastfeeding is associated with a decreased incidence of gastrointestinal (GI) tract infections [1,2], which is corroborated by several studies that have correlated breastfeeding with a lower incidence of necrotizing enterocolitis in humans and animal models [3-5]. Breastfeeding is also associated with an altered fecal microbiome; two studies showed at two weeks of age over 90% of the total fecal bacteria of a breast-fed (BF) infant is *Bifidobacteria*, whereas in most formula-fed (FF) infants *Bifidobacteria* is non-detectable [6,7]. Because the community of gut-colonizing bacteria prevents adhesion and colonization of pathogenic bacteria whilst stimulating mucosal cell proliferation and enhancing immune development, the types of predominant bacteria in the fecal microbiome of the developing infant can affect the health outcomes of the individual, as has been discussed in a recent review article [8]. Human milk, the infant's first food, is a primary source of ingested microbiota. Therefore, it is paramount to fully understand the human milk microbiome and how it might influence colonization of the infant GI tract.

Ingestion of viable bacteria in human milk may lead to effective colonization of the infant GI tract, but the presence of bacterial DNA alone may also hold responsibility for proper infant immune development. For example, unmethylated cytosine phosphate guanine (CpG) dinucleotides within bacterial DNA are known as potent immune stimulators, acting through toll-like receptor 9 [9]. Conversely, immune suppressive motifs including poly-guanosine or guanosine cytosine-rich sequences, such as those on the telomere region of mammalian DNA, that can block immune activation induced by CpGs [10]. Recently, immune suppressive motifs (TTAGGG and TCAAGCTTGA) that are able to counter the effects of CpGs have been discovered in *Lactobacillus*[11]. If immune-modulatory motifs occur in human milk derived DNA, they could contribute to proper immune development by decreasing exaggerated inflammatory responses to colonizing bacteria, which are seen in infants with necrotizing enterocolitis [12].

Human milk bacteria have previously been analyzed by culture-dependent and -independent mechanisms, confirming the presence of a magnitude of bacterial phylotypes [13-20]. In one study, *Staphylococcus* and *Streptococcus* dominated the milk microbiome of most mothers, whereas commercially well known bovine milk-associated genera, *Lactobacillus* and *Bifidobacterium*, contributed as minor milk microbiota members (2–3% of genera) [17]. Another study showed that the human milk microbiome changes over time, and may be dependent on the mother’s weight and the baby’s mode of delivery [20]. Most recent methods for determining the milk microbiome have included amplification of 16S ribosomal RNA genes (rRNA) followed by pyrosequencing [17,20]. Although this technique is widely accepted as a means to determine microbial diversity, it does present limitations such as a lack of information on the functional capacity of the microbes within the milk matrix and also prevents data accumulation on the types of DNA motifs to which an infant is exposed.

In this study we performed a metagenomic analysis of the bacteria in human milk using Illumina sequencing and the MG-RAST pipeline [21]. The aims were to determine the genera of bacteria in human milk, search for immune-modulatory DNA motifs, and determine the types of bacterial open reading frames (ORFs) in human milk that may influence bacterial presence and stability in this complex yet foundational food matrix.

## Results

### Phyla and genera within human milk

**Figure 1:**
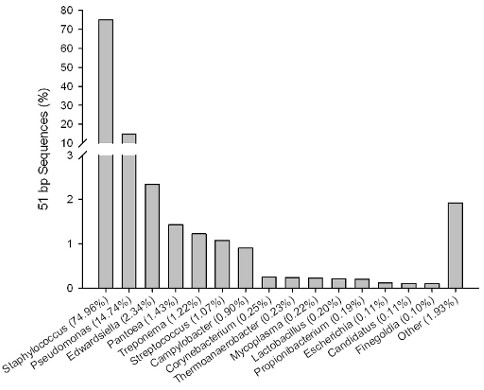
**Best hit analysis of 51 bp DNA sequences from human milk.** DNA from human milk was sequenced using Illumina sequencing followed by alignment to known prokaryotic genomes. Sequences (75,521,216) were BLATed against 1,731 known prokaryotic genomes imported from NCBI (min 95% identity), with 1,331,996 sequences aligning to 370 prokaryotic genera. Other refers to genera each representing <0.1% of all sequences.

Metagenome sequencing of a pooled human milk sample resulted in 261,532,204 sequenced reads of 51 bp, which were binned into those aligning to the human genome (186,010,988, 72.01 ± 3.06%), known prokaryotic genomes (1,331,996, 0.53 ± 0.16%) or those not aligning to either category (74,189,220, 27.46 ± 3.72%, Additional file 1). Using a best hit analysis of the 1,331,996 51-bp sequences, 75% aligned to *Staphylococcus*, 15% to *Pseudomonas*, 2% to *Edwardsiella*, and 1% to *Pantoea*, *Treponema*, *Streptococcus* and *Campylobacter*, respectively (Figure 1). The remaining 3% of the known prokaryotic sequences mapped to 361 bacterial genera, demonstrating the diversity of the human milk metagenome while confirming the presence of key genera like *Akkermansia* (Additional file 2).

**Figure 2:**
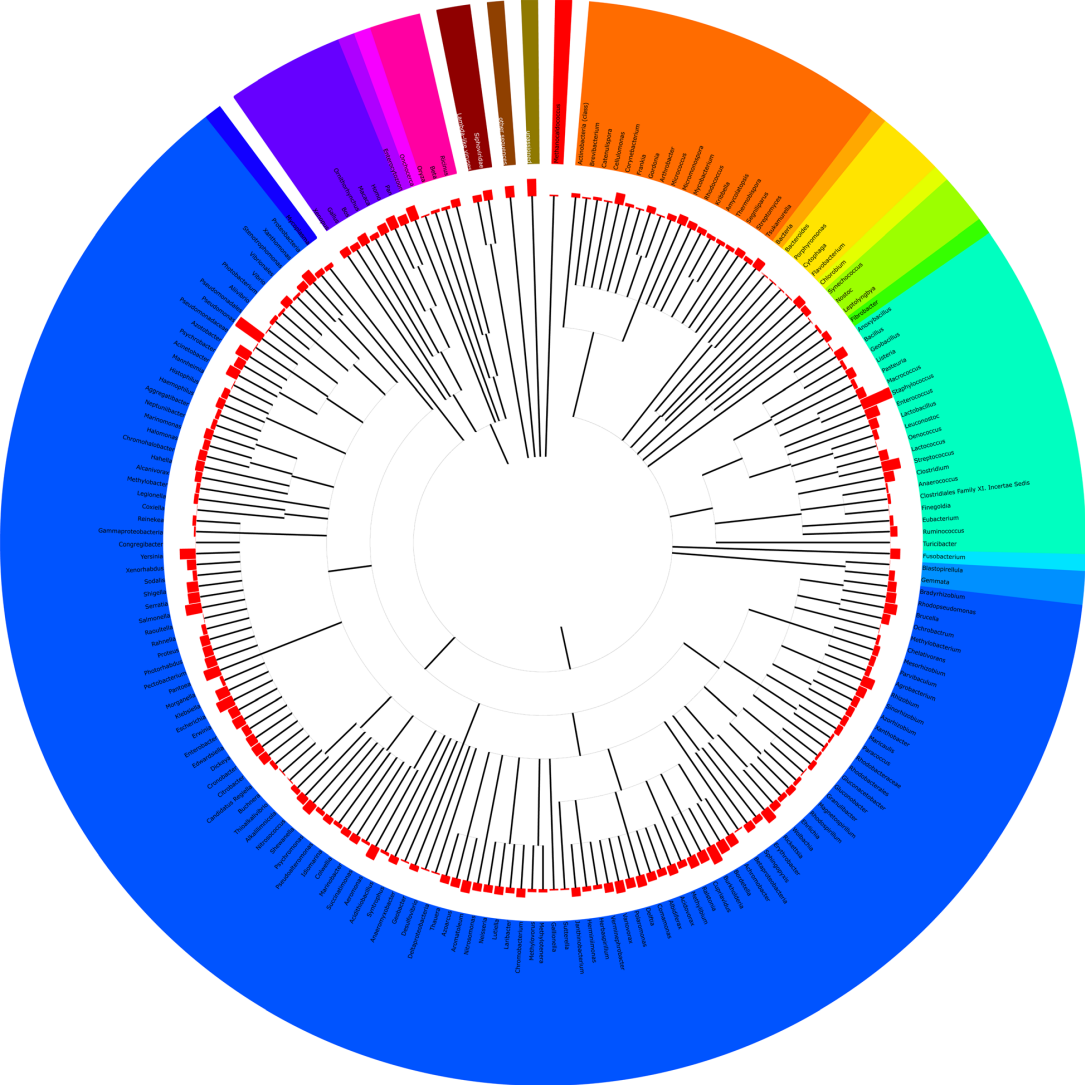
**Best hit analysis of open reading frames within human milk.** Assembled contigs (56,712) were submitted to MG-RAST for analysis. Contigs aligned to 194 known genomes at the genus level (maximum e-value of 1x10^-5^, minimum identity of 60%, and minimum alignment length of 45 bp). Color denotes phylum and red bars indicate the number of positive alignments.

Sequences not aligning to prokaryotic or human genomes with a ≤2 bp mismatch were re-aligned to the human genome with decreased stringency (≤10 bp mismatch), leaving 32,991,450 sequences for contig assembly (Table 1). Using Ray v1.7 [22], 56,712 contigs were assembled and submitted to the MG-RAST pipeline [21]. Post quality control, 53,785 sequences (94.8%), with a mean length of 160 ± 55 bp, were used for further analysis (Table 1). When the contigs were analyzed using a best hit approach through MG-RAST, they aligned predominantly to the phyla of Proteobacteria (65.1%) and Firmicutes (34.6%, Figure 2). The contigs aligned to 194 known genomes at the genus level, predominantly *Pseudomonas* (61.1%), *Staphylococcus* (33.4%) and *Streptococcus* (0.5%), with the highest level of diversity at the genus level within the Proteobacteria phylum (125 different genera, Figure 2). These results are similar to the best hit analysis performed with the non-assembled sequences in that the majority of sequences are from *Staphylococcus* and *Pseudomonas*, but differ in their proportion (Figure 1). Contigs matching viral genomes were observed (<0.04%), including phages derived from *Pseudomonas* and *Staphylococcus* (Figure 2). Contigs also aligned to the genomes of humans, gorillas, chimps and orangutans, likely due to the 60% identity criteria used (Figure 2). The observation of some of the genera, including *Staphylococcus*, *Pseudomonas* and *Pantoea*, was further validated through the presence of their rRNA ORFs (Additional file 3).

**Table 1:** **Contig assembly and open reading frame** (**ORF**) **prediction of Illumina reads** (**51 bp**) **from human milk**

**Sequenced reads(51 bp)**	**261,532,204**
Matching human	186,010,988
Matching prokaryotic	1,331,996
Used in contig assembly^1^	32,991,450
**Contigs**	**56,712**
Post quality control	53,785
Average length (bp)	160 ± 55
Total length (bp)	8,630,997
**Predicted ORFs**	**41,352**
Annotated	33,793
rRNAs	103
Functional category	30,128
Unrecognized	7,559

^1^ all sequences not matching the human genome (≤10 bp mismatch).

### Open reading frames within human milk

**Figure 3:**
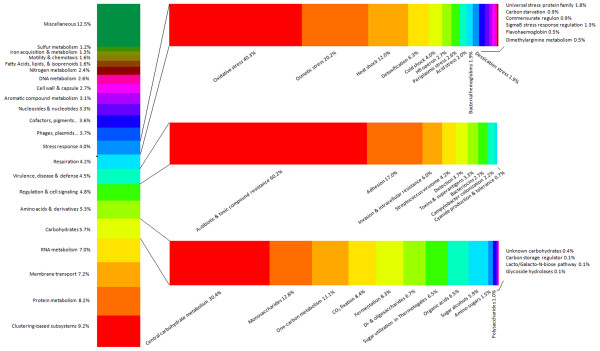
**Functional categorization of open reading frames within human milk.** The percent of ORFs assigned to each functional category is shown. Using the “Hierarchical Classification” tool within MG-RAST, 41,352 ORFs were submitted, 33,793 were annotated and assigned to a functional category (maximum e-value of 1x10^-5^, minimum identity of 60%, and minimum alignment length of 15 aa). Three categories of genes (stress, virulence, carbohydrates) are expanded on the right to demonstrate the diverse capabilities of milk-derived DNA sequences.

A total of 41,352 ORFs were predicted using MG-RAST, of which 82% were annotated (33,793 ORFs), and 18% were unrecognized (7,559 sequences, Table 1). A total of 30,128 ORFs corresponded to a functional category (Figure 3). For example, many ORFs encoded proteins for basic cellular function, including those for respiration (4.2%), cell signaling (4.8%), RNA (7.0%), DNA (2.6%), and amino acid metabolism (5.3%, Figure 3). ORFs encoding proteins for carbohydrate metabolism (5.7% of all ORFs) included those for lactose metabolism (oligosaccharide, 6.7%), but none for human milk oligosaccharide metabolism (Figure 3), likely due to the lack of sequences aligning to the genome of *Bifidobacteria* (Figure 2). Virulence-related ORFs (4.5% of all ORFs) included those for antibiotic resistance (60.2%), adhesion (17%), bacteriocins (2.7%), as well as others (Figure 3). Stress-related ORFs (4.0% of all ORFs) included those for oxidative stress (40.3%), osmotic stress (20.2%), heat and cold shock (12.0% and 4.0%, respectively) and many others (Figure 3).

### Human milk metagenome compared to mothers’ and infants’ feces

**Figure 4:**
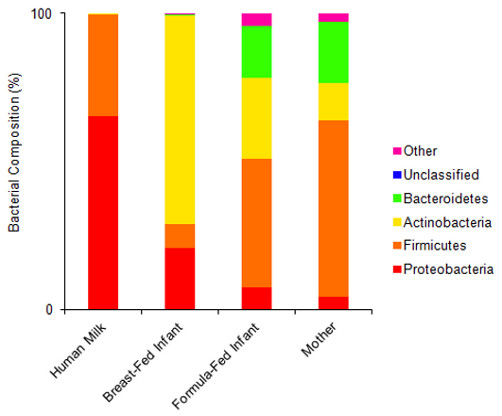
**Best hit comparison of bacterial phyla in human milk**, **infants**’ **feces and mothers**’ **feces.** The percent of sequences assigned to each phyla according to MG-RAST (maximum e-value of 1x10^-5^, minimum identity of 60%, and minimum alignment length of 45 bp) is shown. Breast-fed and formula-fed infant feces values are an average of five individuals, and mothers’ feces values are an average of three individuals. All subjects were unrelated. Other contains phyla each representing <1% of the contigs.

The metagenome of human milk was compared to that of feces from 10 unrelated infants (five BF and five FF) and three unrelated mothers (Figure 4). Using a best hit analysis at the phylum level, contigs from human milk were dissimilar from contigs from feces in regards to the lack of diversity within the human milk metagenome, as over 99% of the contigs were from just two phyla, Proteobacteria and Firmicutes (65.1% and 34.6%, respectively, Figure 4). BF-infants’ feces had a high proportion of Actinobacteria (70.4%), followed by FF-infants’ feces (27.3%), mothers’ feces (12.6%), and human milk (0.15%). The proportion of Proteobacteria in the human milk metagenome (65.1%) was most similar to that of BF-infants’ feces (10.8%), but was significantly different from FF-infants’ feces and mothers’ feces (7.5% and 4.3%, respectively, *P* < 0.05, Figure 2 and Additional file 4). The metagenomes of FF-infants’ feces and mothers’ feces were most similar in regards to their high proportion of Bacteroidetes (17.6% and 20.6%, respectively). Conversely, when using a lowest common ancestor approach at the phylum level in comparison to the best hit analysis, human milk appeared more similar to the fecal metagenomes in terms of an increase in diversity (Additional file 5), but was still dominated by Proteobacteria (38.5%). Also, using the lowest common ancestor analysis increased the proportion of contigs aligning to Actinobacteria in human milk (0.15% to 11.58%), as well as in mothers’ feces (12.6% to 30.6%).

**Figure 5:**
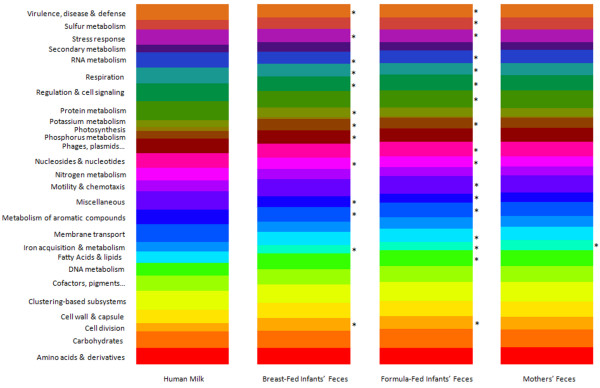
**Functional category comparison of open reading frames within human milk versus infants’and mothers’feces.** The percent of ORFs assigned to each functional category of genes is shown. Using the “Hierarchical Classification” tool within MG-RAST, ORFs within each metagenome were assigned to a functional category (maximum e-value of 1x10^-5^, minimum identity of 60%, and minimum alignment length of 15 aa). Asterisk denotes that the proportion of ORFs within the category is significantly different from that in human milk (Student’s *t*-test, *P* < 0.05). Breast-fed and formula-fed infant feces values are an average of five individuals, and mothers’ feces values are an average of three individuals. All subjects were unrelated.

**Figure 6:**
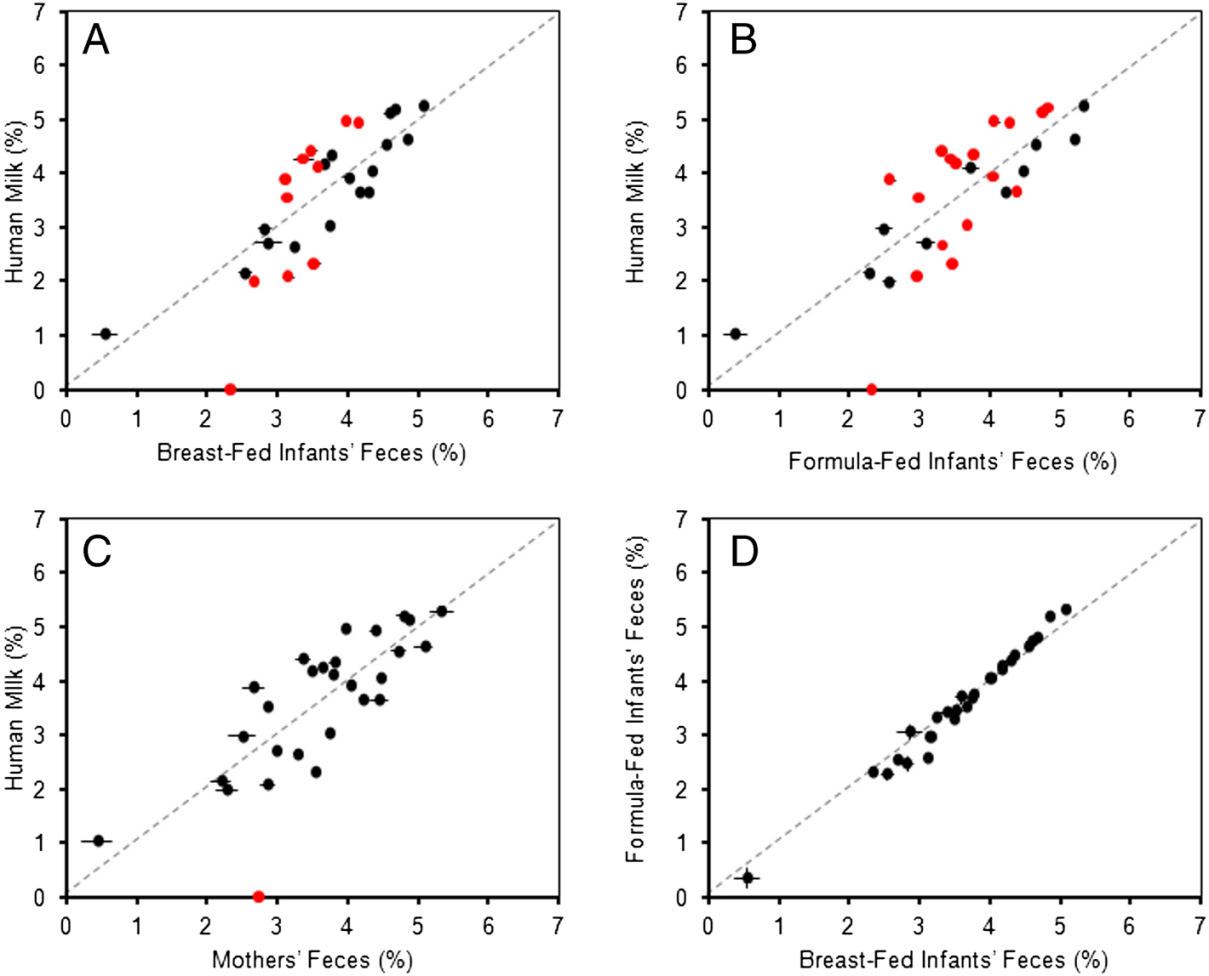
**Pair**-**wise comparison of categorized open reading frames from human milk versus infants’and mothers’feces.** Pair-wise comparisons for the human milk metagenome versus (**A**) breast-fed infants’ feces, (**B**) formula-fed infants’ feces and (**C**) mothers’ feces are shown. For comparison, a plot of breast-fed infants’ feces and formula-fed infants’ feces (**D**) is also shown. Each point represents a different SEED subsystem and its relative abundance within the human milk metagenome compared to the fecal metagenomes. Points lying on or near the dotted line have equal or similar abundance in both metagenomes. Points closer to the x-axis are more abundant in the feces metagenome, whereas points closer to the y-axis are more abundant in the human milk metagenome. Red dots signify those with significantly different proportions between the two metagenomes (Student’s *t*-test, *P* < 0.05). Breast-fed and formula-fed infants’ feces values are an average of five individuals, and mothers’ feces values are an average of three individuals. All subjects are unrelated.

The metagenomes of human milk and feces were also compared at the functional level (Figure 5). The functional ORF profile of the human milk metagenome is similar to that of each fecal metagenome, but two fecal profiles were even more similar, for example BF- versus FF-infants’ feces, as seen using pair-wise comparison plots (Figure 6). The human milk metagenome is most dissimilar from that of FF-infants’ feces as 17 out of the 26 functional categories contain a significantly different proportion of the ORFs (Figure 6). The three fecal metagenomes had a significantly higher proportion of ORFs encoding genes for dormancy and sporulation (2.3%, 2.3% and 2.7%, for BF-infants’, FF-infants’ and mothers’ feces, respectively) than did the human milk metagenome (no associated ORFs, Figures 5 and 6). Both BF- and FF-infants’ fecal metagenomes had significantly higher proportions of cell division (3.5% each, respectively) and phosphorus metabolism related ORFs (3.1% and 3.0%, respectively) than did the human milk metagenome (2.3% and 2.1%, Figures 5 and 6). The human milk metagenome, in comparison to BF- and FF-infants’ feces, did, however, have significantly higher proportions of membrane transport (5.0% compared to 4.0% and 4.0%), nitrogen (3.5% compared to 3.1% and 3.0%) and RNA metabolism (4.9% compared to 4.1% and 4.3%), cell regulation (4.4% compared to 3.5% and 3.3%), respiration (4.3% compared to 3.4% and 3.4%), stress response (4.2% compared to 3.7% and 3.5%) and virulence-related ORFs (4.4% compared to 3.7% and 3.7%, Figures 5 and 6).

### Immune-modulatory DNA motifs in human milk and feces

When contigs were searched for the presence of immune suppressive motifs, TCAAGCTTGA was found in 0.02% of the human-milk assembled contigs (11 sites, Table 2) with an occurrence 1.5 times that of the human genome alone (once per 844,000 bp compared to once per 1,276,500 bp in the human genome, Z-score −1.6). The contigs positive for TCAAGCTTGA aligned to the genomes of *Pseudomonas* (45%), *Nocardia* (9%), *Staphylococcus* (9%) and contigs of unknown origin (36%, Table 3). When the contigs from BF-infants’ feces, FF-infants’ feces and mothers’ feces were scanned for TCAAGCTTGA, it was found at a relative occurrence of 1.19, 1.64, and 1.64 times that in the human genome, respectively (Table 2). Another immune suppressive site, TTAGGG was observed 1,684 times in the human milk metagenome (3.2% of contigs), and at a relative occurrence 0.48 times that of the human genome (once per 5,600 bp compared to once per 2,670 bp, Z-score 22.54, Table 2). Contigs containing TTAGGG corresponded to genomes of *Staphylococcus* (59%), *Pseudomonas* (10%), *Lactobacillus* (0.5%), 21 other known prokaryotic genomes (2.7%), and contigs from unknown genomes (27%, Table 3). When the contigs from BF-infants’ feces, FF-infants’ feces and mothers’ feces were scanned for TTAGGG, this sequence was observed at a relative occurrence of 0.33, 0.18 and 0.26 times that in the human genome, respectively (Table 2). Assembled contigs were also searched for the presence of synthetically-assembled immune suppressive or immune stimulatory DNA motifs (7 and 5 motifs, respectively), such as those used in vaccine production (Additional file 6[23-27]). No synthetically-assembled sequences were observed in the human-milk contigs, whereas three motifs were found in less than 5 × 10^-4^% of contigs from the fecal metagenomes (maximum of 4 hits per 834,774 contigs, Additional file 6).

**Table 2:** Occurrence of immune suppressive motifs in various metagenomes

**Sequence**	**Number of hits**	**Base pairs per hit**		**Relative occurrence** (**Z**-**score**)
**TCAAGCTTGA**	11	844,000	(Human Milk)	1.51 (−1.6)
	344	1,077,000	(BF Infant)	1.19 (−0.74)
	124	779,000	(FF Infant)	1.64 (−1.84)
	268	777,000	(Mother)	1.64 (−1.85)
	2,245	1,276,500	(Human Genome)	
**TTAGGG**	1,684	5,600	(Human Milk)	0.48 (22.54)
	18,118	8,200	(BF Infant)	0.33 (42.54)
	16,410	15,000	(FF Infant)	0.18 (94.85)
	20,612	10,200	(Mother)	0.26 (57.92)
	1,082,623	2,670	(Human Genome)	

DNA motifs TTAGGG and TCAAGCTTGA were searched for in contigs derived from human milk, breast-fed infants’ feces (BF infant), formula-fed infants’ feces (FF infant) and mothers’ feces. Relative occurrence is in comparison to the human genome.

**Table 3:** Occurrence of immune suppressive motifs TTAGGG and TCAAGCTTGA in contigs from human milk

**Sequence**	**Genus**	**Number of hits**
**TCAAGCTTGA**	*Pseudomonas*	5
	*Nocardia*	1
	*Staphylococcus*	1
	Unknown	4
**TTAGGG**	*Staphylococcus*	1000
	*Pseudomonas*	169
	*Lactobacillus*	8
	*Bacillus*	6
	*Streptococcus*	6
	*Streptomyces*	4
	*Tetragenococcus*	4
	Other	25
	Unknown	461

## Discussion

### Genera within human milk

Determining the human milk metagenome, a bodily fluid notably absent from the human microbiome project [28], is crucial for enabling better insight on the process of infant GI colonization and immune development. By pooling DNA from ten human milk samples and subjecting it to Illumina sequencing we have demonstrated the large diversity of the human milk metagenome with over 56,000 contigs aligning to 177 bacterial genera (Figure 2). Previous studies investigating the microbiome of human milk have used both culture-dependent and -independent approaches. Using 16S rRNA sequencing, Hunt et al. have reported several predominant species in human milk including a core of genera found in 15 human milk samples across time: *Streptococcus*, *Staphylococcus*, *Serratia*, *Pseudomonas*, *Corynebacteria*, *Ralstonia*, *Propionibacteria*, *Sphingomonas*, and *Bradyrhizobiaceae*[17]. Other studies showed colostrum was populated mostly by *Weisella* and *Leuconostoc*, followed by *Staphylococcus*, *Streptococcus*, and *Lactococcus*, and that *Akkermansia* were more prevalent in overweight mothers [20,29]. Using a best hit analysis of the 51 bp Illumina reads, alignments for *Akkermansia*, *Propionibacteria*, *Sphingomonas* and *Weisella* were observed (Additional file 2), but because of the small number of base pairs used for the alignment (51 bp) and the lack of assembled contigs associated with these microbes, their presence in our milk samples is a tentative identification. Using PCR-denaturing gradient gel electrophoresis and quantitative PCR, two studies from Martin et al. reported the presence of *Bifidobacterium breve*, *B*. *adolescentis*, *B*. *bifidum* and *B*. *dentium* in human milk, which differs from our findings (Figure 2, [15,16]). This is likely due to the method of DNA extraction used in our study, as we did not incorporate bead-beating as a means to extract DNA from the hard to rupture *Bifidobacterium*[30]. The differences between the previously reported microbial communities and our analysis may also be due, in part, to the geographic location of the mothers, which has been shown to greatly impact the microbiome of individuals [31]. Furthermore, other differences between our characterization of the milk microbiome and those previously reported may be attributed to the means of milk collection. In comparison to previous studies where human milk was expressed from an aseptic breast [13-20], the current method determines the total microbiome (i.e. metagenome) ingested by the infant (from a non-sterilized breast), indicative of what an infant would receive from its mother during suckling.

Because our samples were collected from a non-sterilized breast, it could be hypothesized the human milk metagenome reported here would be similar to that of the skin microbiome. Although no reference database was freely available within MG-RAST for comparison, the metagenome of human milk is similar to previously reported skin profiles in that there is a large proportion of *Staphylococcus*, which is found in moist areas of skin. These moist areas, such as the antecubital fossa (inner fold of the elbow), also contain Betaproteobacteria, such as *Burkholderia* and *Bordetella*, which are present in the milk metagenome (Figure 2[32,33]). The human milk metagenome is also similar to drier areas of the skin such as the plantar heel, which contains Gamaproteobacteria such as *Pseudomonas*[32]. The human milk metagenome is, however, more similar to fecal microbiomes (as described in 16S rRNA studies) due to the large proportion of Firmicutes bacteria within human milk, which is a very minor member of the skin microbiome (Figure 4, [32,33]). Also, the skin of adults tends to contain a high level of *Propionibacteria*, which notably tends to colonize the skin of cesarean-section birthed babies, whereas this genus is minimally represented in our human milk sample using a best hit analysis of the 51 bp Illumina reads (0.2%, Additional file 2, [34,35]). This observation suggests that mother's milk may prove useful as a skin lotion, to re-balance the skin microbiome of C-section babies.

### Phylogenetic differences between human milk and feces

Comparing the metagenome of human milk to that of publicly available infants’ and mothers’ fecal profiles provides insight as to how human milk may lead to proper colonization of the infant gut. When comparing the human milk metagenome to the infant fecal metagenome, there are numerous differences. For example, the metagenome of BF-infants’ feces contains a high proportion of Actinobacteria (70.4%, Figure 4), which correlates with previous studies demonstrating a high abundance of *Bifidobacterium* in the feces of BF-infants whereas FF-infants had a more varied microbiota [6,31,36]. Contigs from human milk, however, aligned mostly with Proteobacteria and Firmicutes (65.1% and 34.6%, respectively, Figure 4). At the phylum level, the present milk metagenome was less diverse than the fecal metagenomes as over 99% of the contigs were from just two phyla, Proteobacteria and Firmicutes (Figure 4). FF-infants’ feces and mothers’ feces were similar in that they both contained contigs aligning to the phylum Bacteroidetes (17.6% and 20.6%, respectively), whereas Bacteroidetes was a very minor component of BF-infants’ feces and human milk (0.3% and 0.02%, respectively, Figure 4). Also, the similar proportion of Firmicutes in human milk compared to mothers’ feces (34.6% and 59.6%, respectively, Figure 4) correlates with the hypothesis that mothers’ milk may be inoculated by immune cells carrying bacteria from the GI tract of the mother to her breast [37-39]. This may be a mechanism by which the human milk microbiome is shaped by the general health of the mother, including her weight [20].

### Functionality of the human milk metagenome

Using Illumina sequencing of all DNA within milk samples permits the prediction of ORFs within assembled contigs and allows for determination of the functional capability of the milk metagenome. A total of 41,352 ORFs were predicted, including those for basic cell function, as well as those that may enable the bacteria to remain in human milk, such as ORFs for carbohydrate metabolism (5.7% of ORFs, Figure 3). The predominant carbohydrate in human milk, lactose, is a potential carbon source for human milk bacteria, and therefore the presence of ORFs associated with its metabolism (6.7% of carbohydrate-associated metabolism, Figure 3) is expected. Another carbon source for bacteria in human milk is human milk oligosaccharides (HMOs), which cannot be digested by the infant [40]. These oligosaccharides, which are heavily fucosylated and readily digested by *Bifidobacteria*, are thought to be responsible for the colonization of BF-infants with high levels of *Bifidobacteria*[41]. Due to a lack of contigs aligning to *Bifidobacteria* (Figure 2), no ORFs encoding genes for HMOs were observed (Figure 3). Recently, HMOs have also been correlated with increased abundance of *Staphylococcus* within human milk, regardless of their inability to utilize the human milk oligosaccharides as a carbon source [42]. The predominance of *Staphylococcus*-aligning contigs in our milk samples supports these findings (Figure 2). Furthermore, there was a significantly higher number of ORFs related to nitrogen metabolism within the human milk metagenome in comparison to BF- and FF-infants’ feces (Figure 5, *P* < 0.05). Because human milk contains 1.48-2.47 g of nitrogen per 100 g of milk, the bacteria within human milk may use it as a nutrient source in addition to lactose and HMOs [43].

Human milk contains an abundance of immune cells, antibodies and antimicrobial proteins (such as lactoferrin, CD14, alpha-lactalbumin, and lysozyme), and therefore the bacteria residing within human milk must harbor mechanisms to combat the milk-endogenous immune system [44-46]. For example, the metagenome of human milk includes ORFs for stress response and defense (4.0% and 4.5% of all ORFs, respectively) including those for oxidative stress (40.3% of stress-related ORFs) and toxic compound resistance (60.2% of defense ORFs, Figure 3). The human milk metagenome also contains ORFs for both heat and cold shock (12% and 4% of stress-related ORFs, Figure 3), which may enable the bacteria to persist in milk post-breast pumping, refrigeration and reheating. This may be of particular importance as human milk banks gain more popularity over time. For example, as described in a recent review by Urbaniak et al., some milk banks deem pasteurization of breast milk unnecessary, while others have an upper limit of 10^5^ organisms per ml [47]. In unpasteurized banked milk and in-home stored milk, if some organisms are able to survive the storage and re-heating process better than others, the bacterial profile of human milk may change to favor better surviving (and not necessarily more beneficial) bacteria. Furthermore, ORFs encoding genes related to virulence and disease (4.5% of all ORFs, Figure 3), are also observed in the human milk metagenome. These ORFs could allow some of the human milk microbes, such as *Staphylococcus aureus*, to cause mastitis in humans when the balance of human milk-antimicrobials to microbes is tilted towards microbial growth [48]. For example, some bacteria within human milk harbor antibiotic resistance genes (60.2% of virulence associated ORFs) allowing them to proliferate regardless of the mother's potential antibiotic use, and some bacteria are able to produce bacteriocins (2.7% of virulence associated ORFs, Figure 3), which could impact the growth of other, less virulent, microbes within the community.

### Immune-modulatory landscape of the human milk metagenome

Because human milk contains a broad spectrum of microbes at the genus level (Figure 2), it likely contributes significantly towards effective colonization of the infant GI tract. In the case of banked human milk, which is Holder pasteurized (65°C for 5–30 min), most bacteria are destroyed, but their proteins and DNA remain [49]. The presence of non-viable bacteria and bacterial DNA in human milk, which are indistinguishable from live bacteria using our approach of DNA isolation and sequencing, may be a way to prime the infant immune system and lead to tolerance of the trillions of bacteria that will inhabit the gut following birth. For example, the immune suppressive motifs, TTAGGG and TCAAGCTTGA [11], are present in 3.0% and 0.02% of the 56,950 human milk-contigs, respectively (1,684 sites, and 11 sites, Table 2). The occurrence of the immune suppressive motifs is similar to that in the metagenomes of BF- and FF infants’ feces, as well as mothers’ feces. This suggests that having a diverse community of microbes may lead to a similar abundance of immune suppressive motifs, regardless of the genera present in the sample. Interestingly, the immune suppressive motif TTAGGG was found in higher abundance in the human genome than in bacterial contigs (one per 2,670 bp in the human genome compared to one per 5,600 bp in the bacterial contigs, Table 2). Colostrum and mature human milk contain between 5 × 10^8^ to 4 × 10^9^ leukocytes/L and between 1 × 10^8^ to 4 × 10^9^ leukocytes/L, respectively, which are mostly macrophages (55%-60%) and neutrophils (30%-40%), with natural killer cells representing up to 12% of the population [50,51]. This suggests that ingestion of the mothers’ DNA, through ingestion of her immune cells and any free circulating DNA may also lead to proper immune development through a balance of concomitant exposure to immune *stimulatory* bacterial CpGs and immune *suppressive* DNA in the mothers’ genome and bacterial genomes.

## Conclusions

Current microbiome studies characterizing the microbial communities of various anatomical niches have revealed vast differences between healthy individuals [28]. These differences can often be attributed to the host’s environment and diet. As demonstrated previously by preliminary 16S rRNA sequencing, the human milk microbiome is similar to other areas of the body in that its composition is unique to each individual [17]. Milk has evolved as the first nutrient source for mammals *ex utero*, with a high level of inter-mother diversity as to the proportions of bacterial genera, immune proteins and nutrients within it [29]. Perhaps, it is the diversity and/or sequences of DNA within the milk metagenome that is beneficial for infants, as opposed to any one specific bacterial genus or species. Recent reviews on human milk outline the phylotypes of bacteria within human milk, but only speculate on the function of the human milk microbiome due to a lack of data on the functional capacity of the microbes within human milk [47,52]. Because of this, we sought to better understand the human milk metagenome on a functional level rather than a solely phylogenetic level. The discovery of the abundance of immune suppressive DNA motifs observed within bacterial and human DNA from human milk, as well as ORFs within the human milk metagenome that allow bacteria to persist in the biological fluid provides a first glance into the functionality of the milk metagenome. Further studies should include those determining the efficacy of milk DNA to modulate the immune system in the GI tract, and a more exhaustive look at the metagenome of human milk and how it relates to infant health outcomes.

### Note added in Proof

During revision of the manuscript, Everard et al published a report suggesting *Akkermansia*, a human mucus colonizer, helps control diet-induced obesity. Everard et al, 2013, Proc Natl Acad Sci USA doi/10.1073/pnas.1219451110.

## Methods

### Donors and sample collection

Breastfeeding women (n = 10) were recruited from the Children’s Hospital of Eastern Ontario (CHEO, Ottawa, Canada) in accordance with the Research Ethics Board of CHEO and the University of Ottawa Research Ethics Board (2007303-01H). Informed consent was given by all participants, all donors were healthy, and milk was donated between 9 and 30 days postpartum. Milk samples were collected by either manual or electric breast pump expression into a sterile milk collection bag (Medela AG, Baar, Switzerland). To better represent a milk sample that would be received by the infant, breasts were not sterilized prior to collection. Samples were immediately frozen and then stored at −70°C.

### DNA isolation

Milk samples (1 mL) were centrifuged at 5,000 × *g* for 10 minutes to pellet eukaryotic cells. Prokaryotic cells were pelleted from milk serum by centrifugation at 13,000 × *g* for 15 minutes. Pellets were resuspended in 2 mL phosphate buffered saline with 1% Triton X-100 and incubated for 2 hours at 37°C to lyse any remaining eukaryotic cells. Bacteria were pelleted by centrifugation at 13,000 × *g* for 15 minutes and pellets were resuspended in 500 μL TE with 30 μL of 10% sodium dodecyl sulfate and 5 μg proteinase K. Samples were incubated for 2 hours at 37°C, and DNA was isolated using phenol/chloroform as previously described [53]. DNA pellets were resuspended in 50 μL TE buffer and pooled. A total of ~4 μg of double stranded DNA was isolated as quantified with Quant-iT PicoGreen (Invitrogen, Burlington, ON, Canada) using a Typhoon Trio Imager and Image Quant TL software (GE Healthcare, Waukesha, WI, USA). DNA integrity was also determined by agarose gel electrophoresis prior to sequencing.

### DNA sequencing, filtering and contig assembly

The pooled DNA sample was sequenced seven independent times by StemCore Laboratories (Ottawa, Ontario, Canada). DNA was prepared according to the DNA sample preparation protocol 1003806 Rev. B for Illumina sequencing (Illumina Inc, San Diego, CA, USA). Sequencing was performed using an Illumina GAIIx Genome Analyzer and Illumina CASAVA analysis pipeline (v 1.7.0). Sequences were aligned to the human genome (hg19/NCBI37) with a stringency of 2 bp mismatching using ELAND (Illumina Inc). Prokaryotic genomes (1,731 genomes) were imported from NCBI. Sequences were aligned to the genomes using BLAT (Kent Informatics, Inc.) and sorted via best hit analysis to genera according to “List of Prokaryotic Names with Standing in Nomenclature” (http://www.bacterio.cict.fr/, accessed February 2012). Unidentified sequences were further filtered by using BLAT against the human genome with a stringency of ≤10 mismatches or gaps. Both prokaryotic and remaining unknown sequences were assembled into contigs using Ray v1.7 [22].

### Contigs, ORF prediction and characterization

Assembled contigs were uploaded to the MG-RAST pipeline [21]. Organism abundance was analyzed using a lowest common ancestor approach with a maximum e-value of 1 × 10^-5^, a minimum identity of 60%, and a minimum alignment length of 15 measured in amino acids for protein and base pairs for RNA databases. A functional abundance analysis of ORFs was performed using "Hierarchical Classification" by comparing to subsystems with a maximum e-value of 1 × 10^-5^, a minimum identity of 60%, and a minimum alignment length of 15 measured in amino acids for protein and base pairs for RNA databases. Previously reported and publicly available metagenomes of feces from five unrelated BF-infants, five FF-infants (metagenome IDs: USinfTW4.1, 6.1, 10.1, 11.1, 12.1, 13.1, 15.1, 19.1, 20.1, and 21.11) and three unrelated mothers (metagenome IDs: USchp [1,3,4,18,33]mom) were compared at the phylum level to the human milk metagenome within MG-RAST using the same lowest common ancestor analysis described above [21]. The mean percent alignments of the individuals were used in Figure 4 and Additional files 4 and 5. The normalized mean percent of ORFs in each functional category was used in Figures 5 and 6. Metagenome comparisons were statistically compared by Student’s *t*-tests (*P* < 0.05) using SigmaPlot (Systat Software, Inc., San Jose, CA, USA).

### Immune-modulatory motif identification

An identity of 100% was used to search for immune-modulatory motifs by alignment with assembled contigs from the human milk metagenome (56,712 contigs) or the fecal metagenomes described above (834,774, 64,662 and 553,391 contigs from BF-infants’, FF-infants’ and mothers’ feces, respectively). The human genome (2,865,822,365 bp) was used as a comparative reference. Z-score was calculated using the formula Z = (O-E)/Stdev, where O was the observed number of hits and E was the expected number of hits using the formula E = (*L*_*cont*_)(N_*h*/_L_*h*_) where *L*_*cont*_ was length of sequences or assembled contigs, N_*h*_ was number of sites found in the human genome (or compiled bacterial genomes); Stdev was the standard deviation of occurrence of each motif in 22 + X + Y human chromosomes.

### Availability of supporting data

The data set supporting the results of this article is available in the MG-RAST repository, under the project name Human_milk_microbiome, http://metagenomics.anl.gov/linkin.cgi?project=2959.

## Abbreviations

BF: Breast-fed; FF: Formula-fed; GI: Gastrointestinal; CpG: Cytosine phosphate guanine; rRNA: Ribosomal RNA; ORF: Open reading frame; HMO: Human milk oligosaccharide; COG: Cluster of orthologous groups.

## Competing interests

The authors declare they have no competing interests.

## Authors’ contributions

TLW designed the data analysis approach, interpreted results and wrote the manuscript. SH performed the data analysis for Figure 1, Tables 1, 2, 3 and Additional file 1: Table S1, Additional file 2: Table S2 and Additional file 6: Table S4. IA conceived and supervised the study and edited the paper. II supervised the bioinformatics analyses and edited the paper. All authors have read and approved the manuscript.

## Supplementary Material

Additional file 1 **Abundance of DNA fragments in pooled human milk, sequenced seven times.** This table contains the number of DNA sequences per run and their general alignments. Click here for file

Additional file 2 **Classification of 51 bp DNA sequences derived from human milk by best hit analysis.** This table contains all genera with at least one alignment match to sequences from human milk-derived DNA. Click here for file

Additional file 3 **Predicted open reading frames from human milk DNA sequences aligning to rRNA genes of known organisms.** This table contains all rRNA genes and their corresponding genera found within the human milk metagenome. Click here for file

Additional file 4 **Pair-wise comparison of phyla abundance in human milk versus infants’ and mothers’ feces metagenomes.** This graph demonstrates the similarities between the human milk metagenome and the fecal metagenomes. Click here for file

Additional file 5 **Lowest common ancestor comparison of bacterial phyla in human milk, and in infants’ and mothers’ feces.** This figure shows the relative abundance of each phylum in the human milk metagenome as compared to the fecal metagenomes. Click here for file

Additional file 6 **Immune-modulatory DNA motifs sought in DNA sequences derived from human milk or feces.** This table shows all synthetically-assembled DNA motifs and their references that were searched for within the human milk and fecal metagenomes. Click here for file
