# Supplementary material for: Human milk metagenome: a functional capacity analysis
Source: BMC Microbiol. 2013 May 25;13:116. doi: 10.1186/1471-2180-13-116 (PMC3679945; doi:10.1186/1471-2180-13-116)
Supplement: Additional file 1 — Abundance of DNA fragments in pooled human milk, sequenced seven times. This table contains the number of DNA sequences per run and their general alignments. [file 1471-2180-13-116-S1.docx]

**Additional file 1.** **Abundance of** **DNA fragments in pooled human milk, sequenced seven times.** Sequences of 51 bp were analyzed by Illumina sequencing and matched to human or prokaryotic genomes (≤2 bp mismatch) by BLAT.

|  | **Sequences per run** | | | |
| --- | --- | --- | --- | --- |
|  | **Total** | **Human** | **Prokaryotic** | **Other** |
|  | 18,193,400 | 14,026,621 | 117,211 | 4,049,568 |
|  | 35,809,228 | 26,466,812 | 211,199 | 9,131,217 |
|  | 50,313,515 | 34,690,538 | 283,882 | 15,339,095 |
|  | 39,021,577 | 27,432,706 | 123,626 | 11,465,245 |
|  | 47,856,320 | 32,191,169 | 142,279 | 15,522,872 |
|  | 27,612,520 | 20,864,809 | 188,516 | 6,559,195 |
|  | 42,725,644 | 30,338,333 | 265,283 | 12,122,028 |
| **Total** | **261,532,204** | **186,010,988** | **1,331,996** | **74,189,220** |
| **Fraction of total ± SE** | | **(72.01 ± 3.06%)** | **(0.53 ± 0.16%)** | **(27.46 ± 3.72%)** |
