# Supplementary material for: Human milk metagenome: a functional capacity analysis
Source: BMC Microbiol. 2013 May 25;13:116. doi: 10.1186/1471-2180-13-116 (PMC3679945; doi:10.1186/1471-2180-13-116)
Supplement: Additional file 2 — Classification of 51 bp DNA sequences derived from human milk by best hit analysis. This table contains all genera with at least one alignment match to sequences from human milk-derived DNA. [file 1471-2180-13-116-S2.docx]

**Additional file 2.** **Classification of 51 bp DNA sequences derived from human milk by best hit analysis.** Each match was characterized with a 95% sequence alignment with the known prokaryotic genome.

| Genus | Percent of Sequences |
| --- | --- |
| Staphylococcus | 74.96 |
| Pseudomonas | 14.74 |
| Edwardsiella | 2.34 |
| Pantoea | 1.43 |
| Treponema | 1.22 |
| Streptococcus | 1.07 |
| Campylobacter | 0.90 |
| Corynebacterium | 0.25 |
| Thermoanaerobacter | 0.23 |
| Mycoplasma | 0.22 |
| Lactobacillus | 0.20 |
| Propionibacterium | 0.19 |
| Escherichia | 0.11 |
| Candidatus | 0.11 |
| Finegoldia | 0.10 |
| Riemerella | 0.09 |
| Bacillus | 0.08 |
| Stenotrophomonas | 0.08 |
| Erwinia | 0.08 |
| Sphingopyxis | 0.08 |
| Caldicellulosiruptor | 0.07 |
| Yersinia | 0.06 |
| Burkholderia | 0.06 |
| Acinetobacter | 0.05 |
| Listeria | 0.04 |
| Mycobacterium | 0.04 |
| Shewanella | 0.04 |
| Klebsiella | 0.04 |
| Brachyspira | 0.04 |
| Azotobacter | 0.04 |
| Agrobacterium | 0.04 |
| Salmonella | 0.04 |
| Enterococcus | 0.04 |
| Enterobacter | 0.03 |
| Verminephrobacter | 0.03 |
| Lactococcus | 0.03 |
| Clostridium | 0.03 |
| Thermoanaerobacterium | 0.03 |
| Pediococcus | 0.03 |
| Buchnera | 0.03 |
| Macrococcus | 0.03 |
| Methanococcus | 0.02 |
| Aeromonas | 0.02 |
| Shigella | 0.02 |
| Bacteroides | 0.02 |
| Cronobacter | 0.02 |
| Ralstonia | 0.02 |
| Borrelia | 0.02 |
| Desulfotomaculum | 0.02 |
| Helicobacter | 0.02 |
| Rothia | 0.01 |
| Xanthomonas | 0.01 |
| Serratia | 0.01 |
| Acidovorax | 0.01 |
| Haemophilus | 0.01 |
| Methanosarcina | 0.01 |
| Neisseria | 0.01 |
| Citrobacter | 0.01 |
| Anaerococcus | 0.01 |
| Legionella | 0.01 |
| Methylobacterium | 0.01 |
| Dickeya | 0.01 |
| Nitrosomonas | 0.01 |
| Geobacillus | 0.01 |
| Veillonella | 0.01 |
| Methanobrevibacter | 0.01 |
| Pectobacterium | 0.01 |
| Rhodothermus | 0.01 |
| Marinobacter | 0.01 |
| Rubrobacter | 0.01 |
| Syntrophomonas | 0.01 |
| Delftia | 0.01 |
| Leptospira | 0.01 |
| Chitinophaga | 0.01 |
| Synechococcus | 0.01 |
| Natrialba | 4.73E-03 |
| Sphingobium | 4.65E-03 |
| Trichodesmium | 4.50E-03 |
| Rhodococcus | 4.35E-03 |
| Micrococcus | 4.28E-03 |
| Spirochaeta | 4.20E-03 |
| Herbaspirillum | 4.20E-03 |
| Lawsonia | 4.13E-03 |
| Chlorobium | 4.05E-03 |
| Bradyrhizobium | 3.98E-03 |
| Methanosalsum | 3.90E-03 |
| Lysinibacillus | 3.83E-03 |
| Flavobacterium | 3.83E-03 |
| Leuconostoc | 3.75E-03 |
| Flavobacteriaceae | 3.75E-03 |
| Cupriavidus | 3.75E-03 |
| Myxococcus | 3.60E-03 |
| Xenorhabdus | 3.53E-03 |
| Pedobacter | 3.53E-03 |
| Oenococcus | 3.53E-03 |
| Alcanivorax | 3.53E-03 |
| Bordetella | 3.45E-03 |
| Azoarcus | 3.45E-03 |
| Rhizobium | 3.38E-03 |
| Halogeometricum | 3.38E-03 |
| Rahnella | 3.30E-03 |
| Polaromonas | 3.30E-03 |
| Ochrobactrum | 3.23E-03 |
| Geobacter | 3.15E-03 |
| Rickettsia | 3.08E-03 |
| Haliangium | 3.08E-03 |
| Brachybacterium | 2.78E-03 |
| Rhodopseudomonas | 2.70E-03 |
| Vibrio | 2.55E-03 |
| Thermodesulfobacterium | 2.55E-03 |
| Leptothrix | 2.55E-03 |
| Sphingomonas | 2.48E-03 |
| Dyadobacter | 2.48E-03 |
| Caulobacter | 2.48E-03 |
| Sinorhizobium | 2.40E-03 |
| Cytophaga | 2.40E-03 |
| Alkalilimnicola | 2.33E-03 |
| Gramella | 2.25E-03 |
| Brevundimonas | 2.25E-03 |
| Granulibacter | 2.18E-03 |
| Prevotella | 2.10E-03 |
| Gardnerella | 2.10E-03 |
| Acidiphilium | 2.10E-03 |
| Arthrobacter | 2.03E-03 |
| Actinobacillus | 2.03E-03 |
| Streptomyces | 1.95E-03 |
| Microbacterium | 1.95E-03 |
| Janthinobacterium | 1.95E-03 |
| Variovorax | 1.80E-03 |
| Pseudoalteromonas | 1.80E-03 |
| Alicycliphilus | 1.80E-03 |
| Pyrolobus | 1.73E-03 |
| Proteus | 1.73E-03 |
| Ramlibacter | 1.65E-03 |
| Cellvibrio | 1.65E-03 |
| Xylella | 1.58E-03 |
| Pseudoxanthomonas | 1.58E-03 |
| Chromobacterium | 1.58E-03 |
| Paracoccus | 1.50E-03 |
| Mobiluncus | 1.50E-03 |
| Marinomonas | 1.43E-03 |
| Laribacter | 1.43E-03 |
| Francisella | 1.43E-03 |
| Maricaulis | 1.28E-03 |
| Herminiimonas | 1.28E-03 |
| Butyrivibrio | 1.28E-03 |
| Xanthobacter | 1.20E-03 |
| Thioalkalivibrio | 1.20E-03 |
| Rhodobacter | 1.20E-03 |
| Frankia | 1.20E-03 |
| Alkaliphilus | 1.20E-03 |
| Halomonas | 1.13E-03 |
| Aggregatibacter | 1.13E-03 |
| Achromobacter | 1.13E-03 |
| Methylomicrobium | 1.05E-03 |
| Dinoroseobacter | 1.05E-03 |
| Bifidobacterium | 1.05E-03 |
| Tolumonas | 9.76E-04 |
| Thauera | 9.76E-04 |
| Psychrobacter | 9.76E-04 |
| Leptotrichia | 9.76E-04 |
| Leadbetterella | 9.76E-04 |
| Pseudogulbenkiania | 9.01E-04 |
| Phenylobacterium | 9.01E-04 |
| Pelobacter | 9.01E-04 |
| Microcystis | 9.01E-04 |
| Kocuria | 9.01E-04 |
| Desulfovibrio | 9.01E-04 |
| Chelativorans | 9.01E-04 |
| Photorhabdus | 8.26E-04 |
| Ferrimonas | 8.26E-04 |
| Exiguobacterium | 8.26E-04 |
| Azospirillum | 8.26E-04 |
| Tetragenococcus | 7.51E-04 |
| Haloterrigena | 7.51E-04 |
| Halorhodospira | 7.51E-04 |
| Aerococcus | 7.51E-04 |
| Pelotomaculum | 6.76E-04 |
| Oceanobacillus | 6.76E-04 |
| Novosphingobium | 6.76E-04 |
| Fusobacterium | 6.76E-04 |
| Deinococcus | 6.76E-04 |
| Thiomonas | 6.01E-04 |
| Prosthecochloris | 6.01E-04 |
| Methanobacterium | 6.01E-04 |
| Saccharopolyspora | 5.26E-04 |
| Porphyromonas | 5.26E-04 |
| Paenibacillus | 5.26E-04 |
| Methanothermobacter | 5.26E-04 |
| Mesorhizobium | 5.26E-04 |
| Hyphomonas | 5.26E-04 |
| Desulfarculus | 5.26E-04 |
| Comamonas | 5.26E-04 |
| Collimonas | 5.26E-04 |
| Clostridiales | 5.26E-04 |
| Beijerinckia | 5.26E-04 |
| Arcanobacterium | 5.26E-04 |
| Sulfolobus | 4.50E-04 |
| Streptobacillus | 4.50E-04 |
| Prochlorococcus | 4.50E-04 |
| Pirellula | 4.50E-04 |
| Pelagibacterium | 4.50E-04 |
| Moraxella | 4.50E-04 |
| Methylibium | 4.50E-04 |
| Methanothermococcus | 4.50E-04 |
| Kytococcus | 4.50E-04 |
| Haloquadratum | 4.50E-04 |
| Gordonia | 4.50E-04 |
| Dechloromonas | 4.50E-04 |
| Clavibacter | 4.50E-04 |
| Ureaplasma | 3.75E-04 |
| Staphylothermus | 3.75E-04 |
| Sorangium | 3.75E-04 |
| Sodalis | 3.75E-04 |
| Roseobacter | 3.75E-04 |
| Rhodospirillum | 3.75E-04 |
| Methylobacillus | 3.75E-04 |
| Hahella | 3.75E-04 |
| Eubacterium | 3.75E-04 |
| Erythrobacter | 3.75E-04 |
| Ehrlichia | 3.75E-04 |
| Chromohalobacter | 3.75E-04 |
| Chloroflexus | 3.75E-04 |
| Capnocytophaga | 3.75E-04 |
| Bartonella | 3.75E-04 |
| Azorhizobium | 3.75E-04 |
| Actinosynnema | 3.75E-04 |
| Thiomicrospira | 3.00E-04 |
| Salinispora | 3.00E-04 |
| Pasteurella | 3.00E-04 |
| Parvibaculum | 3.00E-04 |
| Nocardioides | 3.00E-04 |
| Methylococcus | 3.00E-04 |
| Methanosphaera | 3.00E-04 |
| Methanopyrus | 3.00E-04 |
| Magnetococcus | 3.00E-04 |
| Jonesia | 3.00E-04 |
| Gallibacterium | 3.00E-04 |
| Catenulispora | 3.00E-04 |
| Brevibacillus | 3.00E-04 |
| Arcobacter | 3.00E-04 |
| Anoxybacillus | 3.00E-04 |
| Anabaena | 3.00E-04 |
| Alteromonas | 3.00E-04 |
| Zobellia | 2.25E-04 |
| Uncultured | 2.25E-04 |
| Thermus | 2.25E-04 |
| Thermofilum | 2.25E-04 |
| Streptosporangium | 2.25E-04 |
| Starkeya | 2.25E-04 |
| Roseburia | 2.25E-04 |
| Pusillimonas | 2.25E-04 |
| Parachlamydia | 2.25E-04 |
| Orientia | 2.25E-04 |
| Oligotropha | 2.25E-04 |
| Odoribacter | 2.25E-04 |
| Methylovorus | 2.25E-04 |
| Methanothermus | 2.25E-04 |
| Magnetospirillum | 2.25E-04 |
| Kangiella | 2.25E-04 |
| Jannaschia | 2.25E-04 |
| Hydrogenobaculum | 2.25E-04 |
| Gluconacetobacter | 2.25E-04 |
| Geodermatophilus | 2.25E-04 |
| Desulfobacterium | 2.25E-04 |
| Cellulomonas | 2.25E-04 |
| Carnobacterium | 2.25E-04 |
| Carboxydothermus | 2.25E-04 |
| Beutenbergia | 2.25E-04 |
| Atopobium | 2.25E-04 |
| Aromatoleum | 2.25E-04 |
| Zymomonas | 1.50E-04 |
| Verrucosispora | 1.50E-04 |
| Thermococcus | 1.50E-04 |
| Thermincola | 1.50E-04 |
| Teredinibacter | 1.50E-04 |
| Stackebrandtia | 1.50E-04 |
| Saccharophagus | 1.50E-04 |
| Ruegeria | 1.50E-04 |
| Roseiflexus | 1.50E-04 |
| Psychromonas | 1.50E-04 |
| Polynucleobacter | 1.50E-04 |
| Parabacteroides | 1.50E-04 |
| Oscillibacter | 1.50E-04 |
| Olsenella | 1.50E-04 |
| Nostoc | 1.50E-04 |
| Methylotenera | 1.50E-04 |
| Methylocella | 1.50E-04 |
| Methanospirillum | 1.50E-04 |
| Megasphaera | 1.50E-04 |
| Kribbella | 1.50E-04 |
| Ketogulonicigenium | 1.50E-04 |
| Isoptericola | 1.50E-04 |
| Intrasporangium | 1.50E-04 |
| Halothiobacillus | 1.50E-04 |
| Coxiella | 1.50E-04 |
| Blattabacterium | 1.50E-04 |
| Asticcacaulis | 1.50E-04 |
| Anaeromyxobacter | 1.50E-04 |
| Allochromatium | 1.50E-04 |
| Acidaminococcus | 1.50E-04 |
| Zunongwangia | 7.51E-05 |
| Xylanimonas | 7.51E-05 |
| Weissella | 7.51E-05 |
| Weeksella | 7.51E-05 |
| Waddlia | 7.51E-05 |
| Tsukamurella | 7.51E-05 |
| Thermovibrio | 7.51E-05 |
| Thermosipho | 7.51E-05 |
| Thermobispora | 7.51E-05 |
| Tepidanaerobacter | 7.51E-05 |
| Syntrophobacter | 7.51E-05 |
| Synechocystis | 7.51E-05 |
| Sulfurospirillum | 7.51E-05 |
| Sphingobacterium | 7.51E-05 |
| Sideroxydans | 7.51E-05 |
| Segniliparus | 7.51E-05 |
| Sanguibacter | 7.51E-05 |
| Salinibacter | 7.51E-05 |
| Saccharomonospora | 7.51E-05 |
| Robiginitalea | 7.51E-05 |
| Rhodoferax | 7.51E-05 |
| Renibacterium | 7.51E-05 |
| Pyrococcus | 7.51E-05 |
| Polymorphum | 7.51E-05 |
| Parvularcula | 7.51E-05 |
| Nocardiopsis | 7.51E-05 |
| Nocardia | 7.51E-05 |
| Nitrosospira | 7.51E-05 |
| Nitrosococcus | 7.51E-05 |
| Nitrobacter | 7.51E-05 |
| Nitratifractor | 7.51E-05 |
| Nakamurella | 7.51E-05 |
| Microlunatus | 7.51E-05 |
| Methylomonas | 7.51E-05 |
| Methanosaeta | 7.51E-05 |
| Methanocaldococcus | 7.51E-05 |
| Mesoplasma | 7.51E-05 |
| Meiothermus | 7.51E-05 |
| Mannheimia | 7.51E-05 |
| Lacinutrix | 7.51E-05 |
| Krokinobacter | 7.51E-05 |
| Kosmotoga | 7.51E-05 |
| Kitasatospora | 7.51E-05 |
| Isosphaera | 7.51E-05 |
| Ilyobacter | 7.51E-05 |
| Idiomarina | 7.51E-05 |
| Halomicrobium | 7.51E-05 |
| Haliscomenobacter | 7.51E-05 |
| Fluviicola | 7.51E-05 |
| Ferroglobus | 7.51E-05 |
| Erysipelothrix | 7.51E-05 |
| Elusimicrobium | 7.51E-05 |
| Eggerthella | 7.51E-05 |
| Desulfurivibrio | 7.51E-05 |
| Desulfobacca | 7.51E-05 |
| Cyanothece | 7.51E-05 |
| Croceibacter | 7.51E-05 |
| Chlorobaculum | 7.51E-05 |
| Chlamydophila | 7.51E-05 |
| Brucella | 7.51E-05 |
| Bdellovibrio | 7.51E-05 |
| Archaeoglobus | 7.51E-05 |
| Amycolatopsis | 7.51E-05 |
| Alicyclobacillus | 7.51E-05 |
| Akkermansia | 7.51E-05 |
| Acidimicrobium | 7.51E-05 |
| Acetobacter | 7.51E-05 |
