# Supplementary material for: Human milk metagenome: a functional capacity analysis
Source: BMC Microbiol. 2013 May 25;13:116. doi: 10.1186/1471-2180-13-116 (PMC3679945; doi:10.1186/1471-2180-13-116)
Supplement: Additional file 3 — Predicted open reading frames from human milk DNA sequences aligning to rRNA genes of known organisms. This table contains all rRNA genes and their corresponding genera found within the human milk metagenome. [file 1471-2180-13-116-S3.docx]

**Additional file 3.** **Predicted open reading frames from human milk DNA sequences aligning to rRNA genes from known organisms.** Minimum 99.5% identity and ORF length of 54.

| **Genus** | **Open Reading Frames** |
| --- | --- |
| Pseudomonas | 73 |
| Homo | 40 |
| Pantoea | 30 |
| Staphylococcus | 21 |
| Gorilla | 16 |
| Acholeplasma | 8 |
| Bacillus | 8 |
| Corynebacterium | 8 |
