# Supplementary material for: Human milk metagenome: a functional capacity analysis
Source: BMC Microbiol. 2013 May 25;13:116. doi: 10.1186/1471-2180-13-116 (PMC3679945; doi:10.1186/1471-2180-13-116)
Supplement: Additional file 4 — Pair-wise comparison of phyla abundance in human milk versus infants’ and mothers’ feces metagenomes. This graph demonstrates the similarities between the human milk metagenome and the fecal metagenomes. [file 1471-2180-13-116-S4.docx]

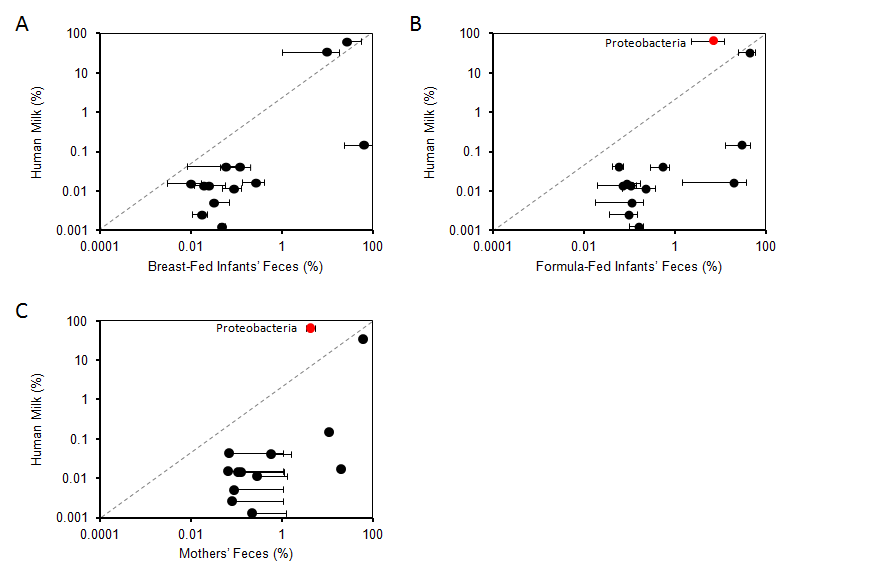


**Additional file 4. Pair-wise comparison of phyla abundance in human milk versus infants’ and mothers’ fecal metagenomes.** Pair-wise comparisons for the human milk metagenome versus (A) breast-fed infants’ feces, (B) formula-fed infants’ feces and (C) mothers’ feces are shown. Each point represents a different phylum and its relative abundance within the human milk metagenome compared to the fecal metagenomes. Points lying on or near the dotted line have equal or similar abundance in both metagenomes. Points closer to the x-axis are more abundant in the feces metagenome, whereas points closer to the y-axis are more abundant in the human milk metagenome. Red dots signify those with significantly different proportions between the two metagenomes (Student’s *t*-test, *P*<0.05). Breast-fed and formula-fed infant feces values are an average of five individuals, and mothers’ feces values are an average of three individuals. All subjects are unrelated.
