# Supplementary material for: Human milk metagenome: a functional capacity analysis
Source: BMC Microbiol. 2013 May 25;13:116. doi: 10.1186/1471-2180-13-116 (PMC3679945; doi:10.1186/1471-2180-13-116)
Supplement: Additional file 5 — Lowest common ancestor comparison of bacterial phyla in human milk, and in infants’ and mothers’ feces. This figure shows the relative abundance of each phylum in the human milk metagenome as compared to the fecal metagenomes. [file 1471-2180-13-116-S5.docx]

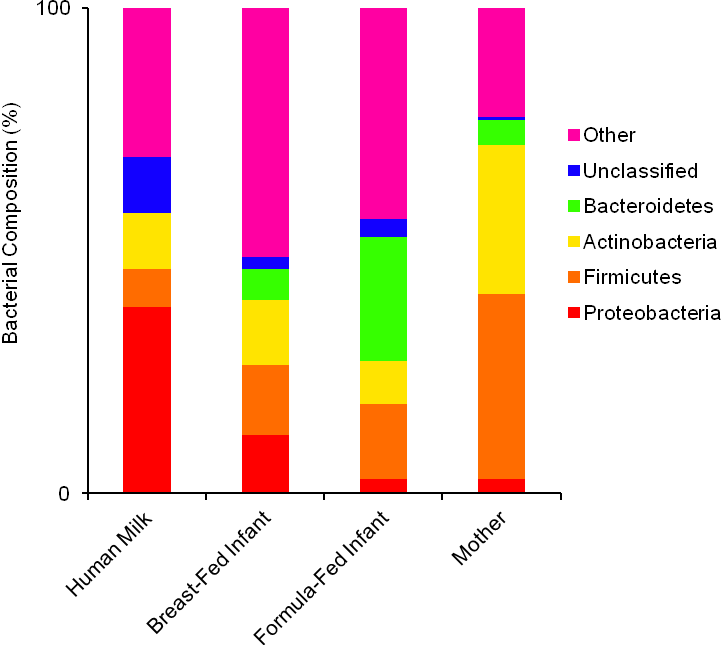


**Additional file 5. Lowest common ancestor comparison of bacterial phyla in human milk, infants’ and mothers’ feces.** Contigs within each metagenomes were assigned to a phyla within MG-RAST (maximum e-value of 1x10^-5^, minimum identity of 60%, and minimum alignment length of 45 bp). Breast fed and formula-fed infant feces values are an average of five samples, and mothers’ feces values are an average of three samples. All subjects are unrelated. Other contains phyla each representing <1% of the contigs.
