# Supplementary material for: Human milk metagenome: a functional capacity analysis
Source: BMC Microbiol. 2013 May 25;13:116. doi: 10.1186/1471-2180-13-116 (PMC3679945; doi:10.1186/1471-2180-13-116)
Supplement: Additional file 6 — Immune-modulatory DNA motifs sought in DNA sequences derived from human milk or feces. This table shows all synthetically-assembled DNA motifs and their references that were searched for within the human milk and fecal metagenomes. [file 1471-2180-13-116-S6.docx]

**Additional file 6. Immune modulatory DNA motifs sought in DNA sequences derived from human milk or feces.** Sequences were searched for in both 51 bp Illumina sequences, as well as in assembled contigs. No motifs listed here were observed in human milk contigs, whereas some were observed in contigs from breast-fed infants’ feces (BF) formula-fed infants’ feces (FF) and mothers’ feces (MF).

| **Motif** | **Observed Hits** | **Immune Modification** | **Reference** |
| --- | --- | --- | --- |
| **TCCATGACGTTCCTGACGTT** | 0 | Suppressive | Zhang X, *et al*. 2012. Shock 38:146-152. |
| **TCCATGACGTTCCTGATGCT** | 0 | Stimulatory | Zhang X, *et al.* |
| **CTCCTATTGGGGGTTTCCTAT** | 0 | Suppressive | Peter M, *et al*. 2007. Immunology 123:118-128. |
| **TCCTGGAGGGGAAGT** | 0 | Suppressive | Ashman RF, *et al*. 2011. International Immunology 23:203-214. |
| **TCCTGACGTTGAAGT** | BF (4), MF (1) | Stimulatory | Ashman RF, *et al*. |
| **TCCTGGAGGGGAAGT** | 0 | Supressive | Ashman RF, *et al*. |
| **TCCTGAAGGGGAAGT** | 0 | Supressive | Ashman RF, *et al*. |
| **TCCTGGCGGGGAAGT** | MF (1) | Supressive | Ashman RF, *et al*. |
| **TCCTGACGGGGAAGT** | FF(1) | Supressive | Stunz LL*, et al.* 2002. European Journal of Immunology 32 :1212-1222. |
| **TCGTCGTTACGTAACGTCGTCGTT** | 0 | Stimulatory | Stunz LL*, et al.* |
| **TCGTCGTTACGTAACGACGTCGTT** | 0 | Stimulatory | Stunz LL*, et al.* |
| **TCGTCGTTCCCCCCCCCCCC** | 0 | Stimulatory | Hartmann G, *et al*. 2000. Journal of Immunology 164:944-952. |
